# “Age” in lung transplantation: factors related to outcomes and other considerations

**DOI:** 10.1007/s13665-016-0151-y

**Published:** 2016-08-13

**Authors:** Christopher H. Wigfield, Vanessa Buie, David Onsager

**Affiliations:** University of Chicago Medicine, Chicago, USA

**Keywords:** Recipient, Donor, Risk, Outcomes, Lung transplantation, Age

## Abstract

The age of lung transplant recipients is steadily increasing. Older donors are more frequently considered. The risk factors associated with advanced age in lung transplantation warrant discussion to ensure optimal outcomes in this complex endeavor. This report provides a summary of the pertinent topics and available evidence.

“Forty is the old age of youth; fifty the youth of old age”.

Victor Hugo

## Introduction

**Fig. 1:**
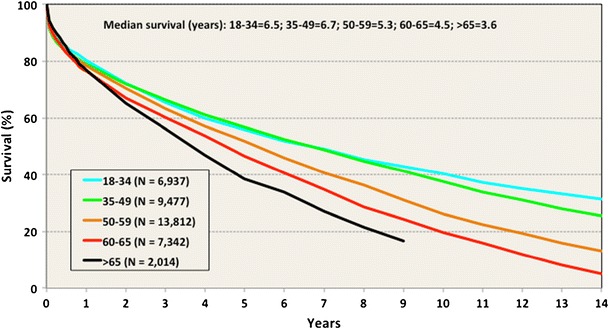
Adult lung transplants. Survival by age group (transplants 1990–2011). Modified from ISHLT Registry [3]. http://www.ishlt.org/registries/slides.asp?slides=heartLungRegistry

Life expectancy is rising in many societies. A growing proportion of the population is over 65 years of age [1, 2]. As a consequence, older patients are referred for consideration for lung transplantation [3]. Prognostic and symptomatic indications exist for this established treatment option in selected candidates with end-stage lung diseases. Lung transplantation, however, remains a complex endeavor and is offered for a wide range of ages with associated risks (see ISHLT data graphic/Fig. 1).

This review provides summary and context for factors contributing to the discussion. Relevant definitions of “age” are offered. The limitations of currently available evidence are recognized. Analyses of registry data and other most relevant information including single center studies and retrospective cohorts are reviewed. The *recipient* age-related concerns are then reviewed. Additionally, we discuss the *donor* age-related allograft problems. Finally, a pragmatic approach to utilize such advanced aged donor lungs in appropriate recipients is considered.

## Background

Age may be defined as a chronological entity or a biological concept. The numerical value ascribed to a person’s age may be a vague indicator, but is frequently seen as insufficient to allow judgment regarding general health status. There is a lack of qualitative understanding in order to allow meaningful care decisions. Biological age, on the other hand, is a complex concept. It entails cell biological processes that are not easily quantified. Age related functionality of organisms has a molecular bases. Fundamentals such as a patient’s tissue regenerative capacity, for example, are not currently measurable. For decisions in clinical practice, it is frequently necessary to compliment the *numeric* age with valid criteria for *functional* age.

**Fig. 2:**
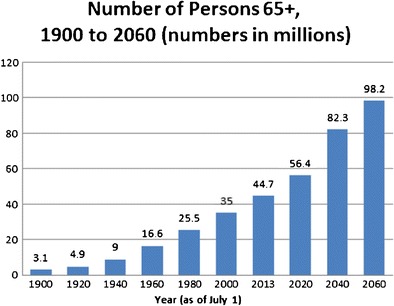
Number of persons 65 years of age or older. 1900–2060 [4]. U.S Census Bureau, Population Estimates and Projections; http://www.aoa.acl.gov/Aging_Statistics/Profile/2014/2.aspx. Accessed July 6, 2015

Assessments of various factors indicative of adequacy of lung transplantation are needed in each potential recipient. Candidates over the age of 70 years are not routinely referred for workup for lung transplantation. Arbitrary restrictions exist for most programs. The conventional definition offered for “elderly” patients is older than 65 years of age (Fig. 2). United Network of Organ Sharing (UNOS) and the International Society of Heart and Lung Transplantation (ISHLT) offer pertinent registry data [4, 5]. These suggest a steady shift toward older recipients over the last decade. Several centers have reported successful transplantation in patients with markedly more advanced ages.

Compared to other solid organ transplants, lung transplantation outcomes are recognized as still suboptimal [6••]. Yet, a liberation of inclusion criteria has occurred over the last decade [7••]. Outcomes observed, in turn, ultimately influence the allocation of allografts. This is particularly pertinent in view of the persistent shortage of adequate donor lungs. The appropriateness of lung transplantation at older ages remains a matter of debate (Fig. 1). Age, therefore, has become a focus of concern in many programs. Although not unique to lung transplantation, both recipient age-related challenges and donor age-associated factors are frequently under intense scrutiny.

### Phenomena related to aging

It is generally agreed that the numeric age of some patients may not reflect their “biological” age or performance status. Determining age in biological systems, however, is a complex task. Even theoretical considerations are multifaceted. Four related phenomena have to be considered: the *process* of aging, the *determinants of longevity*, *age-associated diseases* encountered, and *death* per se [8]. The application of biological aging to the context of lung transplantation needs to acknowledge multiple clinical confounders and nonmedical lifetime limitations that pertain to individual patients.

A distinct set of genetic disorders associated with DNA repair mechanisms provided evidence regarding the need of functional genome maintenance during the aging process [9]. Bio-gerontologists have specified the determinants of aging. These are not simply driven by genetic factors. Ageing is the result of the “loss of molecular fidelity” and ensuing cellular and metabolic dysfunction [10, 11]. The concept of antagonistic pleiotropy describes the beneficial role of particular genes early in life at the expense of inducing “aging” later in life [12]. Telomere changes are an area of intense research interest. Phenotypical consequences of aging, however, may not always be visible. In turn, inherited dysfunction or mutations may affect telomerases without correlation of chronological age [13].

External, i.e., environmental, triggers impact cellular regulation and immunogenic phenotype changes. The sum of these molecular processes leads to cellular and organismal senescence. Additionally, stress response research provides new insights into the profound alterations of normally phylogenetically preserved biochemical signaling pathways [14]. The example of the live-span shortening effect of excessive caloric intake and other specific nutritional deficits has been well described. These are cumulative in the process of ageing due to the metabolic disorders caused.

The process of ageing related general physiologic changes have been subject to considerable research efforts. Regarding lung transplant candidates, the pulmonary manifestations and systemic performance are of primary concern. Pulmonary manifestations of advancing age may aggravate any underlying respiratory disease process. The net effect may be that earlier referral for lung transplant evaluation is required. Specifically, reduced pulmonary elasticity and dependent zone airway closures are likely in the elderly. In addition, chest wall stiffness and loss of muscle mass add restrictive elements to the respiratory impairment. Physiological correlates include a reduced forced expiratory volume (FEV1) and increased residual volume, a decline in vital capacity as well as total lung capacity. Chest wall restriction and body mass index-related issues would then affect the recipient in the post-transplant phase even in the presence of a functional allograft [15, 16].

Other aging-related cellular adaptations have a more wide-ranging impact. Mitochondrial dysfunction and reduced respiratory metabolism with increased deleterious effects of reactive oxygen radicals develop. Multiple alterations of the immune system have been documented in the elderly. A decline of the adaptive immune responses and specific changes of the innate immune-system are well documented. Cellular immunity may be affected by reduced T cell subpopulations, impaired B cell generation, and non-organ-specific auto-antibody formation [17]. Susceptibility to certain pathogens has been demonstrated and hyper-reactivity of airways may develop.

Age-related specific diseases then add to the burden with individual end-organ manifestations including central nervous system and neuro-muscular and degenerative impairment. The resulting net effect of markedly reduced physiological reserve may not be apparent until a response to critical illness stressors is required. Therefore, a plethora of age-related possible comorbidities must be considered and actively investigated in elderly candidates for transplantation. Some patients will present with absolute contraindications defined for most lung transplant programs, due to excessive perioperative risks perceived and the ability to recover adequately post-operatively (Table 1). Other age-related disease processes are clearly identifiable with screening and manageable with adequate specialist care systems in place. As the incidence of cancer increases with age, evaluation for specific risk factors and application of screening protocols for the most common malignancies is an essential part of the assessment of elderly candidates for potential lung transplantation.

**Table 1:** Examples for age-related comorbidities

Possible age-related comorbidities in older lung transplant candidates	
Systemic disease	Malignancies, hypertension, cardiovascular, diabetes mellitus, nutritional deficits, falls, and injuries
CNS disease	Memory impairment, CVA, Parkinson’s disease hearing loss, vision issues, sleep disorders
End-organ disease	Coronary artery disease, thyroid disease, urinary disorders, arthritis, osteoporosis, GI disorders, pressure ulcers

Additional concerns implicit in caring for elderly patients are the risk of failure to thrive and other systemic comorbidities difficult to reverse [18]. These include anorexia of aging and relative cognitive impairment that require detailed clinical assessment and at times specialist evaluation for underlying causes. Similar to general frailty, these entities are hard to quantify. They demand clinical acuity and judgment in the absence of reliable biomarkers.

### Lung transplantation in the elderly

Life expectancy varies among societies and socioeconomic circumstances. Currently, expected remaining years of life in the US for a person reaching the age of 65 is 17.9 years for males and 20.5 years for females [19]. Lung transplantation survival rates are frequently reported for multiple age groups. National survival rate averages reported for lung transplantation in the US by UNOS are 82 % at 1 year, 65 % at 3 years, and 51 % at 5 years, respectively. The primary motivation in lung transplants is to improve the 1-year mortality observed due to their underlying respiratory disease. Few elderly recipients, however, will have a fully restored “life expectancy”. It is therefore critical to understand each individual’s expectations in life and consider the symptomatic benefit for most of these patients undergoing lung transplantation.

Despite the chronological continuum, registries report outcomes observed after lung transplantation in arbitrarily selected age categories. Such analysis often considers older recipients (>60 years of age and separately > 65 years). The ISHLT registry and UNOS data provide such retrospective information regarding transplant rates and survival outcomes in these cohorts [5, 19]. This cannot include biological variances of patients at different ages and has to be seen as general indicators rather than meaningful for individual patient assessment.

The age of recipients, when assessed according to the era of lung transplantation in the ISHLT registry, also showed a marked increase over time for 60–65- and >65-year-old recipients [20••]. This trend of increasing median age for lung transplant candidates has not been affected by implementation of the Lung Allocation Score (LAS) in the US [21]. The proportion of the 60–69-year-old recipients in particular has steadily increased over the last 15 years according to UNOS 2014 data.

Single-center reviews provided limited evidence for survival outcomes in “older” patient cohorts. These studies used variable age cutoffs and are not directly comparable with meta-analysis [22]. Notable in some of these cohorts are the higher incidence of lethal infective complications in such older recipients. Reviewing the complication rates of elderly recipients, Tomascek et al. reported specific patient characteristics associated with impaired outcomes in a single center review. These included postoperative complications, length of stay, achieved FEV1, and development of BOS at 1 year. BMI was identified to impact overall complication rates in their cohort older than 60 years at time of transplantation [23]. Body composition and frailty have not been considered in these reviews. No useful biomarkers or surrogate parameters were available at the time of these retrospective studies.

**Fig. 3:**
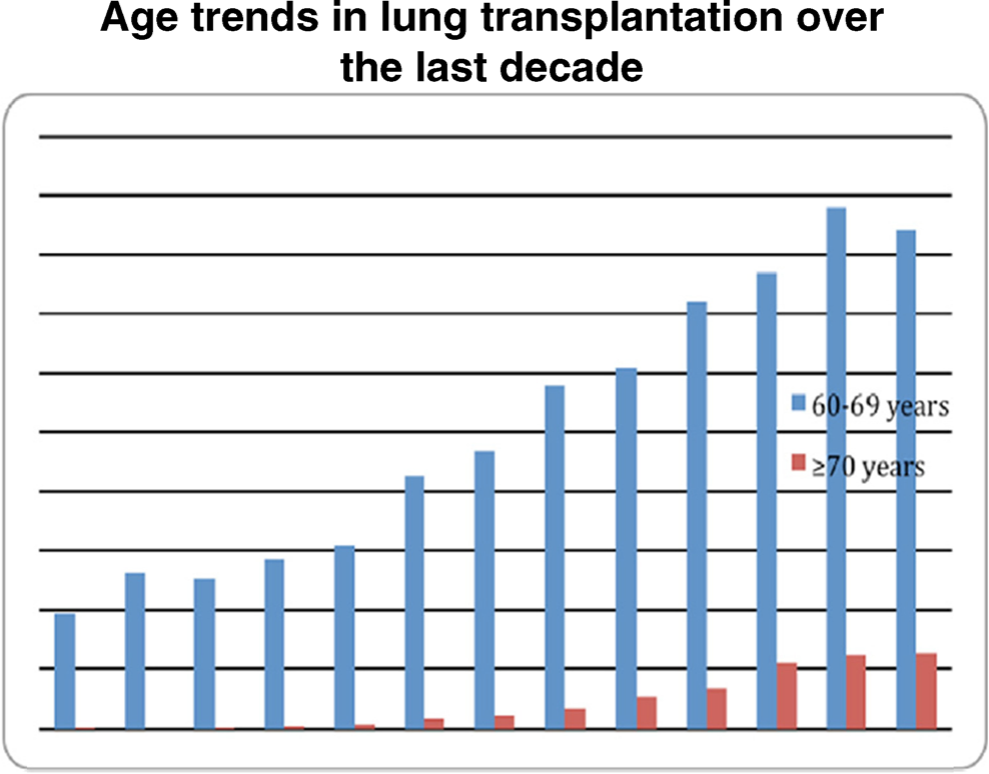
Trends in lung transplantation over the period of 2000 to 2012 for septuagenarians and sexagenarians in the USA, modified from Hayanga et al. [24]

A report reviewing available SRTR data indicates that 1-year survival for septuagenarians in lung transplantation is significantly worse when compared with 60–69-year-old recipients in the cohort prior to 2005; (57 vs. 76 % 1-year survival, log rank *p* = 0.01) [24]. Three- and 5-year survival was noted to be significantly less for elderly lung transplant recipients. There appears to have been an improvement of survival in septuagenarians since the LAS inception in the USA (Fig. 3). This may be manifest despite increased proportion of idiopathic pulmonary fibrosis in this cohort. The cohort comprised 225 recipients with an observed one-year survival of 79 %. Due to their relative improvement, advanced age was not considered to be an “absolute” contraindication [24].

A comparison of older (defined as >60 years of age) to younger recipients in the Toronto Lung Transplant program published in 2007 showed a marked 1-year survival difference (60 vs. 86 %). A difference in survival outcome was also noted at 5 years (37 vs. 57 %; *p* = 0.005). The small number of complete 5 years follow-up for recipients was among the limiting factors in this and other similar single center studies published to date. Infectious complications were the predominant cause for early deaths noted in this study. It also noted the excess mortality for elderly recipients despite adjustment for the Declining Exponential Approximation to Life Expectancy (DEALE) in the older recipient group [23].

Vadnerker et al reviewed age-related complications in recipient older than 65 years in 2011. A higher incidence of malignancy and acute cellular rejection requiring augmented immunosuppression was detected in this particular cohort compared with the institutional experience of 60–65-year-old recipients [25]. The associated editorial by Rahman et Cahill points out that there was no detectable short-term survival difference in this recent cohort with an overall relatively high rate of complications. The lack of disease-specific analysis and long-term survival of elderly recipients is apparent.

Case reports provide limited insight into current practice of individual lung transplant programs. Shigemura et al. reported lung transplantation for a single octogenarian with good early outcome but concede that “A number of complicated issues remain debatable, including ethical concerns…” Clearly, the appropriate allocation of suitable allografts as a scarce commodity remains a concern.

A more recent analysis of the ISHLT registry data compared the median survival of recipients separated into five age groups. The Kaplan–Meier survival curves provided to show a marked separation after 12 months for recipients older than 60 years old. There is a further attrition rate apparent when comparing the subgroup older than 65 years old (*n* = 2014) with all other recipient age groups (age groups in years <34, *n* = 6937; 35–49, *n* = 9477; 50–59, *n* = 13812; 60–65, *n* = 7342). This represents the best registry evidence available to guide transplant programs. The retrospective nature of the analysis limits the value for individual case management, as it does not adjust for other selection criteria. The precise impact of age on prognostic value of lung transplantation would require prospective studies with surrogate markers for age-related conditions in this context.

### Donor age impact in lung transplantation

The dichotomy of the transplant process is evident when considering the donor-related circumstances. The prevalence of pulmonary diseases among the ten leading causes of death must be recognized. Ruling out pulmonary malignancy, chronic obstructive airway disease and pneumonia may not be simple in the donor setting. Furthermore, viral respiratory tract infections have seasonal variation with higher prevalence in senior donors with little clinical correlation available.

It is noteworthy that currently no regulatory restrictions are imposed on acceptance of older donor lungs. Individual lung transplant programs generally follow selection criteria protocols. Current lung allocation systems do not incentivize procurement of older donor lungs and utilization rates remain low. Nevertheless, the impact of numerous donor parameters has been studied to assess the possible impact on outcomes after lung transplantation [26, 27]. The donor age variable features in several retrospective analyses.

Satisfactory outcomes can be achieved with older lung allografts, but the impact of age on physiologic changes of lung function must be appreciated [15]. Decreased elasticity with resulting residual volume increase and vital capacity decrease may be a concern. Such parameters and likely increased A-a O_2_ gradients of older lungs may not be quantifiable in the procurement situation. Potential chest wall stiffness and muscle mass reduction of older donors, however, may not have a clinically important impact on total lung capacity and maximum inspiratory pressures, as they arguably depend more on the recipient’s condition in the peritransplant period.

In biological terms, the immunology of advanced age donor lungs warrants some attention.  The decline of adaptive immune system function and the changed state of the innate immune responses will affect the lung allograft. These changes are not quantifiable in current routine procurement practice but may render older allografts to higher susceptibility to particular respiratory tract infections and therefore increase risks as inherited by the recipient [17]. Donors of advanced age have been shown to express lower levels of interleukin-10 early after reperfusion. Cytokine gradients in turn are known to correlate with the development of primary graft dysfunction. The risk of primary graft dysfunction in the absence of rejection and cardiogenic issues or infective pathogens causing graft impairment has been studied.

Although early outcomes of utilization of older lungs have been shown to be similar to those in recipients of lungs from younger donors, late survival and the development of bronchiolitis obliterans syndrome have been negatively impacted. There is currently no correlation of different donor age groups to the various chronic lung allograft dysfunction (CLAD) phenotypes observed.

The influence of donor and recipient age in lung transplantation was studied by Hayes et al. with a review of the United Network of Organ Sharing (UNOS) SRTR registry data. There is evidence that the number of older donor’s allografts being utilized for transplantation is increasing [5]. Multivariate Cox models applied to the large cohort demonstrated that donors below 50 years of age or over reduced the recipients mortality if less than 60 years old at time of transplantation. If, however, the recipient was over the age of 65, only donors less than 50 years old decreased the risk for death after transplantation. The primary finding was that older donor lungs appeared not to affect survival when transplanted for older recipients but limited outcomes in “younger recipients.” Such data suggests that in order to optimally utilize the donor pool, older donor lungs should be used but some *donor-recipient age matching* should be considered. This approach may potentially enhance long-term outcomes in specific recipient age subgroups. It presents an additional challenge in the complex matching of allografts. After exclusion of significant functional impairment, and in addition to serology, immunology, and size, age similarity in older could be advocated in such recipients.

“Age-range matching” allocation of older lung allografts (>60 years old) may provide a pragmatic approach to increase the utilization rate of these organs offered but frequently rejected. A recently published “focus theme age” in lung transplantation analyzed the information available in the ISHLT registry. Two-thirds of recipients are aged 45–65 years, but the trend for 60–69 years old and older than 70 years old is increasing (see Fig. 3). Extremes of age in recipients confer an independent risk factor for early death after transplants. The model utilized in this retrospective analysis also demonstrates older donor age to be an independent risk factor associated with mortality after lung transplantation [20••].

## Conclusions

Age will increasingly factor in the decision-making process for lung transplant candidates. When considering patients for the rigors of lung transplantation, life expectancy must not be confused with individuals’ expectations in life. Frequently, patients are seeking symptom relief and the known prognostic limitations germane to lung transplantation may appear acceptable to older candidates. Informed consent obtained from such candidates ought to include a specific benefit versus risk discussion. For established subgroups of patients with end-stage respiratory disease who have a reasonable survival advantage with transplantation, the systemic aspects inherent with aging cannot be ignored.

The reported 1-year outcomes available from SRTR and ISHLT registries are currently the best source of information for generic decisions. Future analyses ought to further study the impact of age on outcomes in lung transplantation. The overriding concern, to allocate scarce lung allografts to the most appropriate recipients, has to include the assessment of the patient’s potential for full recovery, irrespective of “chronological” age at the time of transplantation. Three-year survival outcomes observed in the subpopulation transplanted at age 65 or older remain inferior to those observed for younger age categories. Lungs from donors with a chronological age of 45 and older can safely be transplanted, but the stakes may be higher for such allografts especially in combination with other extended donor criteria.

The advent of ex vivo lung perfusion systems (EVLP) may allow for detailed differential assessment of older donor lungs. This may lead to separation and selective use of “good old lungs.” Interventions aimed at regenerative or repair mechanisms may be feasible to improve lung procurement to optimize aged allografts.

A key concern remains who may be the most appropriate recipient for any given organ to enhance outcomes. The matching of older donors with advanced age recipients may be a reasonable way of increasing utilization rates of such allografts available. This may help to a modest extent to alleviate the lung allograft shortage and serve the increasingly listed and carefully selected elderly candidates for lung transplantation.

In light of the current evidence available, we advocate for *age matching* of older donor lungs to optimize utilization with a pragmatic approach.
